# Atezolizumab-Induced Thyroiditis and Immune Checkpoint Inhibitor (ICI)-Type 1 Diabetes Mellitus: Diagnostic and Therapeutic Challenges in ICI-Associated Endocrinopathies

**DOI:** 10.7759/cureus.94593

**Published:** 2025-10-14

**Authors:** Rafal Al-Rubaye, Sabha Nadeem, Ahmed Abdulkader Zaki Ali Soliman

**Affiliations:** 1 Acute Medicine, The Shrewsbury and Telford Hospital NHS Trust, Telford, GBR; 2 Internal Medicine, The Shrewsbury and Telford Hospital NHS Trust, Telford, GBR; 3 Endocrinology and Diabetes, Royal Stoke University Hospital, Stoke on Trent, GBR

**Keywords:** atezolizumab, dka, immunotherapy, small cell lung cancer(sclc), thyroiditits

## Abstract

The rapid evolution and increased application of immune checkpoint inhibitors (ICIs) in the oncology setting have introduced novel diagnostic and therapeutic challenges, particularly in the setting of endocrine immune-related adverse events (irAEs). Thyroid dysfunction is among the most common of these, while ICI-type 1 diabetes mellitus is uncommon and can present abruptly with life-threatening complications. A 76-year-old man with extensive-stage small-cell lung cancer being treated with atezolizumab developed new-onset diabetes, which manifested as diabetic ketoacidosis in connection with immunotherapy-induced thyroiditis. Metabolic instability in this patient was compounded by the inappropriate initiation of thyroid hormone replacement during the thyrotoxic phase of the thyroiditis as a result of misinterpretation of his thyroid function tests (TFTs). The unique clinical course of this patient illustrates the broad and unpredictable spectrum of ICI-related endocrinopathies and highlights the importance of prudent interpretation of TFTs, early recognition of ICI-induced type 1 diabetes mellitus, and close multidisciplinary management of irAEs.

## Introduction

Immune checkpoint inhibitors (ICIs) are now an integral part of contemporary cancer treatment and have been shown to improve survival across several tumor types, including small-cell lung cancer [1]. Atezolizumab is a monoclonal antibody that targets programmed death-ligand 1 (PD-L1) and is approved in combination with carboplatin and etoposide as first-line treatment for extensive-stage small cell lung cancer, based on improved survival seen in the phase III IMpower133 trial compared with chemotherapy alone [2].

As their use becomes more widespread, ICIs have been associated with a broad range of immune-related adverse events (irAEs), which are thought to represent non-specific immune activation [3]. Endocrine irAEs are amongst the most frequently reported, often involving the thyroid, pituitary, pancreas, or adrenal glands [4]. Thyroid dysfunction is the most frequently observed endocrine irAE, affecting approximately 20-40% of patients receiving immune checkpoint inhibitors. It typically presents as thyrotoxicosis, hypothyroidism, or a transient thyroiditis that may evolve through both phases. [5,6]. In contrast, ICI-induced type 1 diabetes mellitus is rare (<1%) but clinically important given its acute presentation and tendency to present with diabetic ketoacidosis (DKA) [7].

The presence of multiple endocrine irAEs in the same patient has been less frequently reported and can present significant diagnostic and therapeutic challenges. In particular, misinterpretation of thyroid function tests can lead to unnecessary and potentially harmful interventions and may worsen metabolic instability. We present a patient with small-cell lung cancer treated with atezolizumab who developed new-onset type 1 diabetes mellitus presenting with DKA in the context of immunotherapy-induced thyroiditis and subsequent adrenal insufficiency.

## Case presentation

A 76-year-old gentleman with a medical history of metastatic extensive-stage small cell lung cancer (T4N3M1c), with lung and adrenal metastases, under active treatment with atezolizumab for the past six months and stable prostate adenocarcinoma on hormonal therapy, presented with one week of worsening polydipsia, polyuria, vomiting, and weight loss. He was found to have diabetic ketoacidosis (DKA) in the setting of new-onset type 1 diabetes mellitus (DM1), as he had no prior history of diabetes. His blood glucose and ketone levels were found to be ‘off the scale’. HbA1c was 64 mmol/mol, significantly higher than 35 mmol/mol eight months prior.

Baseline admission blood tests demonstrated a sodium of 128 mmol/L, potassium 5.0 mmol/L, urea 14.1 mmol/L, and creatinine 115 μmol/L (eGFR 53 mL/min/1.73m²). The results were in keeping with stage 1 acute kidney injury (AKI). The hyponatremia was felt to be a pseudohyponatraemia in the setting of severe hyperglycemia. He was managed with the hospital DKA protocol (intravenous insulin and fluid therapy), which was subsequently converted to a basal bolus insulin regimen on resolution of the DKA.

**Figure 1:**
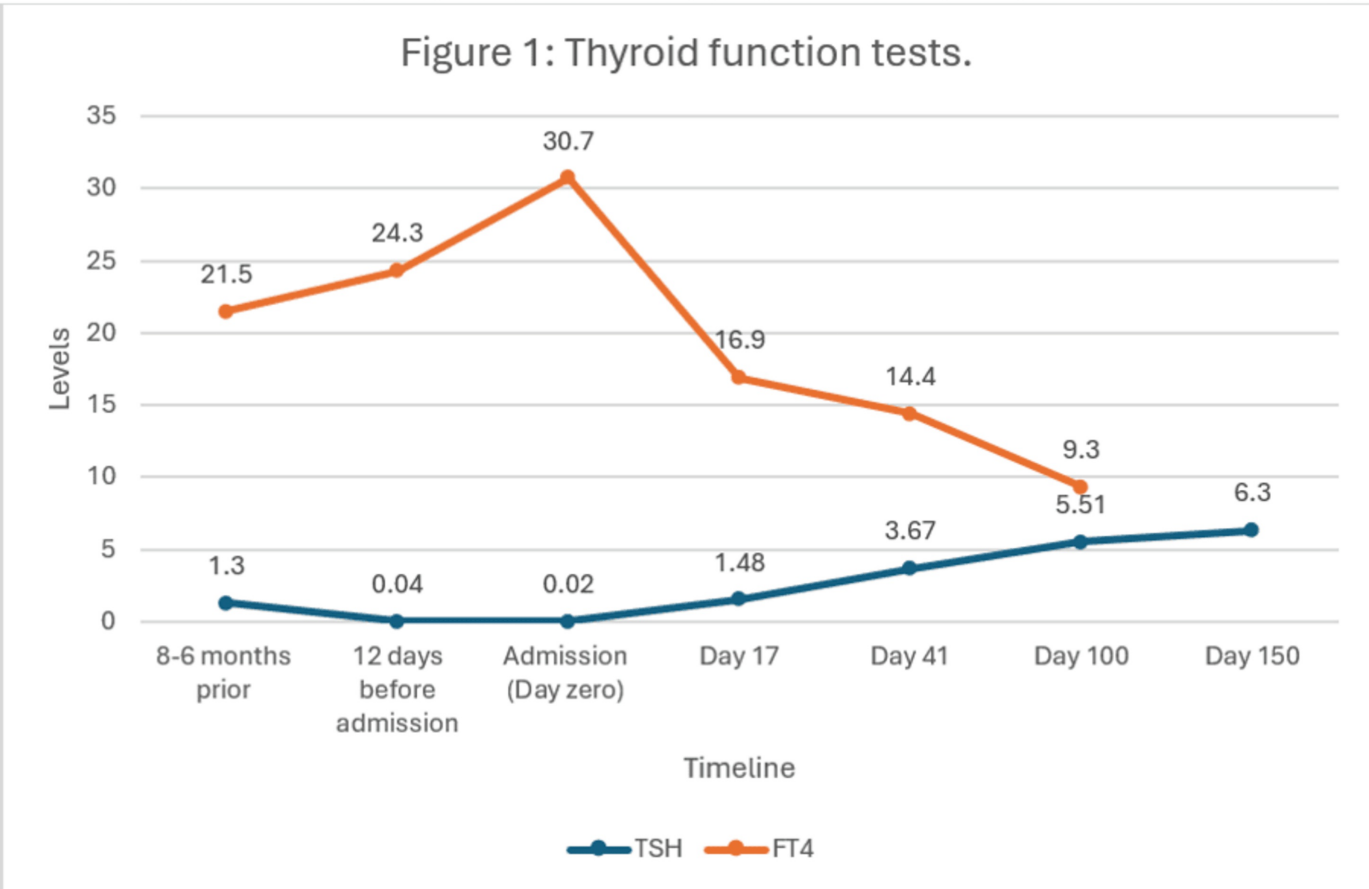
Thyroid function tests

Thyroid function tests (TFTs) were also documented to be abnormal during the admission, and the patient was referred to the endocrinology team. Review of TFTs one month previously revealed a thyroid-stimulating hormone (TSH) of 0.04 mU/L and free thyroxine (FT4) of 23.4 pmol/L, which was consistent with thyrotoxicosis. It was noted that the patient had been erroneously commenced on Levothyroxine 75 μg daily 12 days before admission, pending TFTs results, based on the oncology consultant clinically suspecting the diagnosis of hypophysitis and secondary hypothyroidism in the setting of the low TSH (awaiting FT4 at time of prescription which, if reported before Levothyroxine was prescribed, would likely have prevented the further worsening of hyperglycaemia and precipitation of DKA). Repeat TFTs on admission confirmed further TSH suppression (0.02 mU/L) and an increase in FT4 (30.7 pmol/L) (Figure 1).

Examination post-DKA resolution demonstrated the patient to be clinically well. Observations were stable [early warning score (EWS) 1; pulse rate (PR) 83 bpm, blood pressure (BP) 111/61 mmHg]. The patient was euvolemic. The patient was not tremulous, and there was no palpable thyroid enlargement. The patient did not report palpitations, diarrhea, or heat intolerance.

In addition to TFTs and basic bloods, a panel of autoimmune screening for type-1 diabetes and autoimmune thyrotoxicosis was performed to aid in determining the etiology of the patient’s new-onset diabetes mellitus and thyroid dysfunction. Thyroid peroxidase (TPO) antibodies were within normal limits (titers too low to detect), suggesting no underlying thyroid autoimmune pathology. Anti-glutamic acid decarboxylase (GAD) antibody, IA-2 autoantibody, and Zinc transporter 8 (ZnT8) antibody were all tested, and results were negative, excluding traditional markers of autoimmune type 1 diabetes (Table 1, antibody panel).

**Table 1:** Blood tests FSH: follicle-stimulating hormone; LH: luteinizing hormone; ACTH: adrenocorticotropic hormone; IGF-1: insulin-like growth factor 1; PSA: prostate-specific antigen; GAD: glutamic acid decarboxylase; IA2: islet antigen-2;

Blood test		Normal range
FSH	Normal	
LH	<1.0 iu/L (low)	1-10 iu/L
Prolactin	402 mu/L (high)	80-330 mu/L
ACTH	Normal	
Serum IGF-1	1.5 nmol/L (low)	5-25 nmol/L
Cortisol	30 nmol/L (low)	
PSA	0.07 ug/L	0-6.50 ug/L
Thyroid peroxidase antibody	<15 iu/mL	0-34 iu/L.
Anti-GAD antibodies	Negative	
IA2 Auto antibodies	Negative	
Zinc trans 8 antibodies	Negative	
C peptide	111 pmol/L	

C-peptide was 111 pmol/L, consistent with marked suppression of endogenous insulin production. The absence of diabetes-related antibodies, in the context of low C-peptide levels and new onset of diabetes following immune checkpoint inhibitor (ICI) treatment, is highly suggestive of ICI-induced type-1 diabetes mellitus.

The endocrinology team was also able to determine that the patient had developed immunotherapy-induced thyroiditis in the thyrotoxic phase. Levothyroxine was stopped, and since the patient was asymptomatic from thyrotoxicosis, he was discharged with follow-up in the endocrinology outpatient clinic. He was subsequently followed up in the endocrinology clinic for his thyroid function. The course of his thyroid status is shown in Figure 1.

It was noted that around one month following discharge, TSH began to come out of suppression, reflecting the transition out of the thyrotoxic phase. Six months following his original admission, TSH levels had started to be raised above the reference range, in keeping with the development of the hypothyroid phase of immunotherapy-induced thyroiditis. In contrast to this, his FT4 levels had shown a different trajectory, with a gradual decline, returning to the normal range at around four months, earlier than the rise in TSH levels. This reflects the typical biphasic clinical course of ICI-related thyroiditis, with an initial thyrotoxic phase, followed by a subsequent hypothyroid phase.

Immunotherapy was stopped after five months, as the patient went on to develop further endocrinopathies. In addition to immunotherapy-induced thyroiditis and type 1 diabetes mellitus, he was later diagnosed with adrenal insufficiency (Table 1, showing a low cortisol level), which, in combination with the other endocrinopathies, was suggestive of an evolving polyendocrinopathy. In view of this, ICI therapy was ceased.

## Discussion

Endocrine irAEs are now recognized as an important and increasingly common complication of ICI therapy. In contrast to most other irAEs, endocrinopathies are typically permanent, reflecting irreversible glandular damage and hence require hormone replacement rather than immunosuppression in most cases [8]. Thyroid dysfunction is the most common endocrine irAE, while ICI-type 1 DM is rare, though potentially life-threatening [6,9]. The coexistence of multiple endocrine irAEs in the same individual, as in this case, is certainly rare, but is well recognized in the literature and creates specific diagnostic and management challenges.

The incidence of ICI-DM has been estimated at around 0.5%, with a recent population-based study reporting an incidence of approximately 125/100,000 person-years, much higher than in the general population [9,10]. DKA on presentation is seen in up to 70-80% of cases, reflecting the abrupt and fulminant nature of the β-cell destruction [11,12]. This clinical phenotype is further associated with very low or undetectable C-peptide levels (as in our patient), although classical autoimmune type 1 diabetes is associated with islet autoantibodies in the majority of patients, only around 40-50% of ICI-DM cases have detectable antibodies, so antibody negativity is not a diagnostic exclusion [11,12]. Taken together with the temporal relationship between ICI exposure and the development of abrupt insulin dependence in this patient, it is hard to conceive of an etiology other than ICI-DM.

In contrast to ICI-DM, thyroid irAEs are much more common, being seen in 8-22% of patients receiving PD-1/PD-L1 inhibitors [6]. These typically follow a biphasic pattern of transient thyrotoxicosis, in keeping with destructive thyroiditis, before eventually developing hypothyroidism [7,13]. The trajectory of thyroid function tests in this patient, with suppressed TSH and elevated FT4 on admission, followed by recovery, then rise of TSH above the normal limits with time and eventual development of hypothyroidism, is typical of this natural history. Management of the thyrotoxic phase is generally supportive, with β-blockade if required, and starting levothyroxine is usually deferred until the hypothyroid phase [13].

A particularly important learning point in this case was the inappropriately early initiation of levothyroxine in the thyrotoxic phase. The suppressed TSH was misinterpreted as central hypothyroidism, leading to initiation of thyroid hormone replacement before FT4 was available. It is a fundamental tenet of thyroid physiology that TSH should never be interpreted in isolation, and guidelines emphasize the need to review FT4 to avoid such errors [8]. In this case, levothyroxine likely has contributed to the worsening of hyperglycemia and precipitated DKA. It serves as a timely reminder of the clinical pitfalls of isolated reliance on TSH in isolation in oncology practice, and the need for careful endocrinology input in the management of ICI-related thyroid dysfunction.

Although hypophysitis is another well-recognized endocrine irAE, its incidence is much lower with PD-1/PD-L1 blockade compared with cytotoxic T-lymphocyte-associated antigen 4 (CTLA-4) inhibitors, and when it occurs, it usually presents as central adrenal insufficiency with low or inappropriately normal TSH and FT4 [14,15]. In this case, suppressed TSH with elevated FT4 was much more consistent with thyroiditis, which the later evolution into hypothyroidism with raised TSH level confirmed.

The subsequent development of adrenal insufficiency in this case raises the possibility of evolving polyendocrinopathy. Although less frequently described than in our case, simultaneous or sequential involvement of multiple endocrine glands is increasingly recognized in the setting of ICIs [16]. It emphasizes the need for ongoing vigilance and longitudinal monitoring across endocrine axes once any single irAE has occurred.

Finally, it remains a source of debate whether ICIs should be continued or discontinued in the presence of endocrine irAEs. Current consensus states that the majority of endocrine toxicities can be managed with hormone replacement alone without the need to permanently withhold immunotherapy [8]. In the case of multi-gland involvement, recurrent crises such as DKA or progressive toxicity, temporary or even permanent interruption may be necessary. In this patient, the development of diabetes, thyroiditis, and adrenal insufficiency led to a decision to discontinue atezolizumab to allow stabilization of endocrine function. This reflects the importance of individualized, case-by-case management decisions, ideally within a multidisciplinary team, in order to optimize the balance of oncological benefit versus risk of cumulative endocrine morbidity.

In conclusion, this case highlights the at times unpredictable spectrum of ICI-associated endocrinopathies. It emphasizes the importance of careful biochemical interpretation, in particular avoidance of misclassification of thyroid status, and the need for close monitoring across multiple endocrine axes. As with all irAEs, early endocrinology involvement is important, and multidisciplinary care is key to optimizing both oncological and endocrine outcomes.

## Conclusions

In this case, the patient had received atezolizumab which had led to radiological improvement with complete resolution of adrenal metastases and improvement in pulmonary nodules; such improvement is consistent with a significant partial response.

With cessation of immunotherapy in the setting of progressive endocrine-related adverse events, the patient has developed radiological progression with development of intracranial metastases. This patient has now been referred for palliative cranial radiotherapy in an attempt to preserve neurological function and provide optimal symptom control.

The overall prognosis in this patient is guarded given both disease stage and recent development of central nervous system metastases. Care is being provided in a multidisciplinary fashion with efforts being made to stabilize endocrine dysfunction.
